# Using Feedback Control to Reduce Limb Impedance during Forceful Contractions

**DOI:** 10.1038/s41598-017-10181-9

**Published:** 2017-08-24

**Authors:** Xiao Hu, Daniel Ludvig, Wendy M. Murray, Eric J. Perreault

**Affiliations:** 1 Departement of Biomedical Engineering, Northwestern University, Evanston, IL USA; 2 Sensory Motor Performance Program, Rehabilitation Institute of Chicago, Chicago, IL USA; 3 Departement of Physical Medicine and Rehabilitation, Northwestern University, Chicago, IL USA; 4 Research Service, Edward Hines, Jr. VA Hospital, Hines, IL USA

## Abstract

Little is known about the ability to precisely regulate forces or torques during unexpected disturbances, as required during numerous tasks. Effective force regulation implies small changes in force responding to externally imposed displacements, a behavior characterized by low limb impedance. This task can be challenging, since the intrinsic impedance of muscles increases when generating volitional forces. The purpose of this study was to examine the ability to voluntarily reduce limb impedance during force regulation, and the neural mechanisms associated with that ability. Small displacement perturbations were used to quantify elbow impedance during the exertion of volitional elbow torques from 0% to 20% of maximum voluntary contraction. Subjects were instructed either to not intervene with the imposed perturbations or to explicitly intervene so as to minimize the influence of the perturbations on the elbow torque. Our results demonstrated that individuals can reduce the low frequency components of elbow impedance by 35%. Electromyographic analysis suggested that this behavior is mediated by volitional and possibly long-latency reflex pathways with delays of at least 120 ms. These results provide a context for understanding how feedback altered by aging or injuries may influence the ability to regulate forces precisely.

## Introduction

Numerous tasks require the precise control of force. Consequently, there have been many studies aimed at understanding the ability to maintain steady forces, and the physiological mechanisms contributing to that ability^1–6^. While these studies have yielded many insights, they have not considered how force is regulated during unexpected disturbances, as is relevant to the many real-world tasks that involve interaction with objects that have uncertain properties or are being manipulated in an uncertain environment. The objective of this work was to examine the ability to maintain a constant force during unexpected perturbations of arm posture.

Effective force regulation implies that external perturbations of limb posture should result in only small changes in the force being produced. This is equivalent to stating that the limb has low impedance, since impedance describes the dynamic relationship between externally imposed displacements of a limb and the forces generated in response^7^. For small perturbations applied under steady state conditions, limb impedance can be approximated by the inertia, viscosity and stiffness of the limb^7, 8^. For high frequencies, corresponding to rapid postural perturbations, limb inertia is the most dominant component of impedance. In contrast, stiffness is the primary contributor for lower frequencies corresponding to slow or even static postural displacements. Muscle and limb stiffness increase with increasing levels of activation^9–13^. This provides a simple mechanism to maintain a desired posture in the presence of unexpected disturbances^14–16^, but it would inevitably limit the ability to keep limb stiffness low, as is desirable during force regulation tasks.

Feedforward and feedback strategies can be used to compensate for the increase in muscle stiffness that occurs with increasing muscle force, thereby decreasing interaction forces with the environment. A feedforward strategy can be used to minimize muscle co-contraction such that only the stiffness of the agonist muscles contributes to the net impedance of the limb^17^. Feedforward control may also be useful for reducing the change in force that results from predictable perturbations by appropriately tracking the anticipated disturbance^18^. Though this strategy can be used to reduce interaction forces for predictable perturbations, it would not be appropriate for unpredictable perturbations as accurate motion tracking typically leads to increased limb stiffness^14^. Hence, while feedforward strategies are certainly appropriate for certain tasks, they cannot be used to reduce limb impedance below what can be attributed to the intrinsic force-dependent stiffness of the muscles within the limb, which is necessary for force regulation under unpredictable perturbations.

Feedback mechanisms are needed to compensate for intrinsic muscle properties under unpredictable perturbations. Though much is known about the peripheral sensors that would enable force regulation^19^, the neural pathways processing this information remain uncertain in the force regulation task. Mugge, *et al*.^20^ suggested that short-latency spinal reflexes (~30 ms delay) could be used to lower ankle impedance, but their results were based on modeling the mechanical properties of the ankle rather than direct measures of the neural responses. Others have suggested that compensatory responses are first seen at times corresponding to long latency reflexes (~70 ms)^21–23^, though the relationship between these responses and the net impedance of the limb has not been fully quantified or compared with the longer latency responses that extend into the volitional time period (~150 ms).

No matter which mechanisms contribute to the feedback regulation of force, neural delays are likely to limit their efficacy. Indeed, Mugge *et al*.^20, 24^ demonstrated that only the low-frequency impedance of the ankle could be regulated in a task-dependent manner. These studies, however, only considered the regulation of ankle impedance when the net volitional force was zero, whereas the simultaneous generation of volitional forces is required for many interaction tasks. In addition to the intrinsic stiffness of muscle, stretch reflexes opposing external perturbations of posture also increase with volitional activation, potentially further increasing the stiffness of the limb and reducing the ability to regulate force precisely^25–27^. Hence, it remains unclear to what extent humans can reduce limb impedance during tasks that require active force generation.

The purpose of this study was to quantify the ability to voluntarily reduce limb impedance during force regulation tasks, and to assess the neural mechanisms underlying this ability. This was accomplished by quantifying elbow impedance during the exertion of volitional torques of 10% and 20% of maximum voluntary contraction (MVC), as well as for zero net torque (0% MVC) about the joint. Elbow impedance was estimated using small, stochastic position perturbations. Two tasks were considered: a “do not intervene” (DNI) task, in which subjects were instructed not to respond to the perturbation, and a “torque control” task, in which subjects were instructed to keep the elbow torque constant even when perturbed, thereby attempting to volitionally reduce elbow impedance. We hypothesized that the low frequency component of elbow impedance would be lower during the torque control task. Electromyographic (EMG) responses to the perturbations were used to relate changes in the ability to regulate elbow impedance to the underlying changes in muscle activation, and to estimate the delays in the associated neural control. Together, our results demonstrate the degree to which humans are able to behave as an ideal torque controller, and provide insight to the neural mechanisms contributing to that ability.

Portions of this work have been presented previously in abstract form^28^.

## Results

### Efficacy of Elbow Torque Control

**Figure 1:**
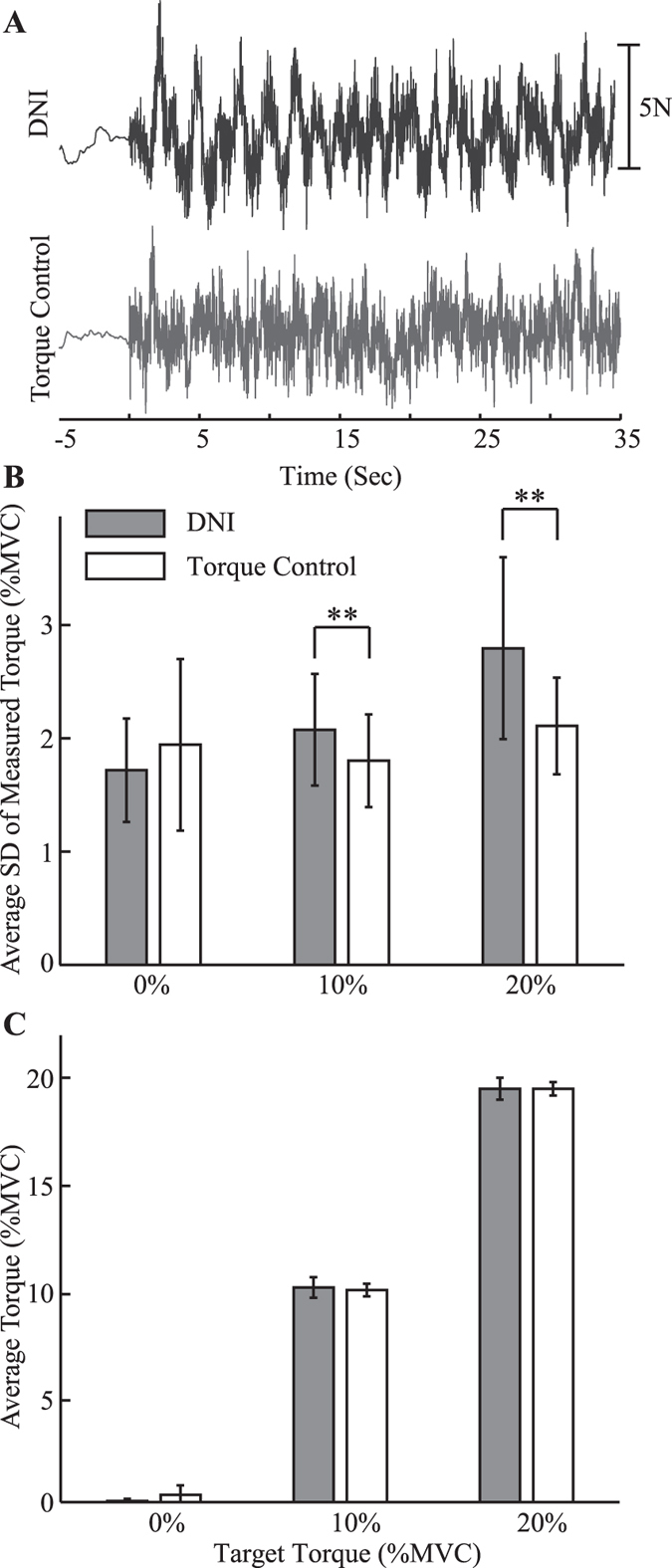
Task-dependent change in elbow torque. (**A**) Raw torque trajectories from subject S5. The subject was exerting a torque of 10% MVC during the DNI task (upper trace) and the torque control task (lower trace). (**B**) The comparison of the average standard deviation (SD) for the torques measured in each task. (**C**) Comparison of the average torques for all subjects during the two tasks performed at 0%, 10% and 20% MVC. Error bars indicate standard deviations. The asterisks **correspond to a significant difference (*P* < 0.01).

Subjects were able to complete the torque control task, reducing the change in elbow torque due to the applied perturbations relative to that measured during the DNI tasks. This was observed by the reduction in the amplitude of the measured torque response to the applied perturbation (Fig. 1A). Across all subjects, the standard deviation in the torque control task was significantly lower than that in the DNI task for mean torque levels of 10% (*F*_1,9_ = 14.5, *P* = 0.0042) and 20% (*F*_1,9_ = 14.7, *P* = 0.0040) of MVC (Fig. 1B). There were no corresponding differences in the mean torque level between the torque control and DNI tasks (*F*_1,9_ = 1.4, *P* = 0.27 at 10% MVC; and *F*_1,9_ = 0.0, *P* = 0.99 at 20% MVC; Fig. 1C). The standard deviation of the torque measured for the 0% MVC target was not significantly different between the two tasks (*F*_1,9_ = 3.1, *P = *0.11; Fig. 1B).

**Figure 2:**
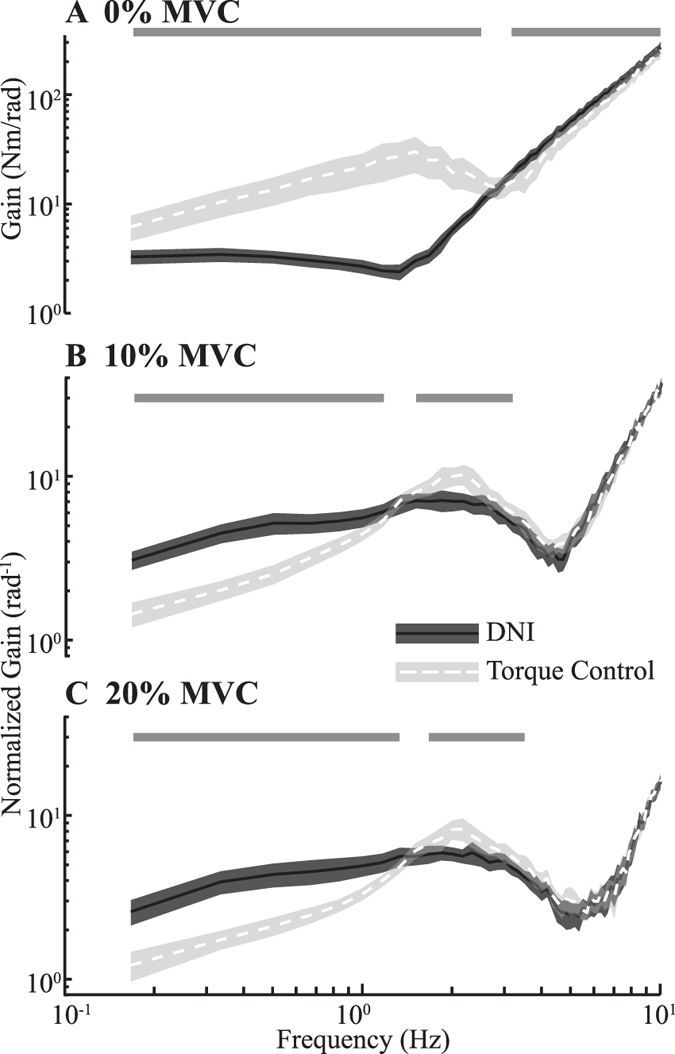
Group average of impedance transfer functions estimated in the DNI (solid curve) and the torque control (dashed curve) tasks. (**A**) Average transfer function for 0% MVC trials. (**B**) Normalized average transfer function for 10% MVC trials. (**C**) Normalized average transfer function for 20% MVC trials. The transfer function for each subject was normalized by the target torque prior to averaging. Note that this was not done in (**A**) since the target torque was zero. The bars on the top of each panel indicate the range of frequencies for which the gain of estimated transfer functions differed significantly (*P* < 0.05) between the torque control and the DNI tasks. The shaded areas indicate 95% confidence intervals.

The ability to complete the torque control task was further demonstrated by reduced elbow impedance relative to that estimated in the DNI tasks. At low frequencies below approximately 1 Hz, impedance magnitude was 35 ± 12% (mean ± 95% confidence interval) smaller in the torque control task than in the DNI task when subjects exerted voluntary torques of 10% MVC; it was 40 ± 13% smaller when performing torque control at 20% MVC (Fig. 2B and C). In contrast, the impedance in this same range was 65 ± 21% smaller in the DNI task than the torque control task performed at 0% MVC (Fig. 2A). There was also a small but consistent increase in the elbow impedance between approximately 2-4 Hz during the torque control tasks performed at 10% and 20% MVC. In the high frequency range (above 4 Hz), there was no significant difference in the elbow impedance between two tasks at 10% and 20% MVC, since the gain of transfer function was dominated by the inertia of the arm, which stayed the same in two tasks. There was a small but statistically significant difference within this range for the tasks performed at 0% MVC, possibly because voluntary attempts to control a torque of zero would have required alternating flexion and extension, thereby resulting in small movements within the cast that would not have been present in the tasks that had a bias in the flexion direction.

### Feedback Contributions to the Regulation of Elbow Impedance during Torque Control Tasks

**Figure 3:**
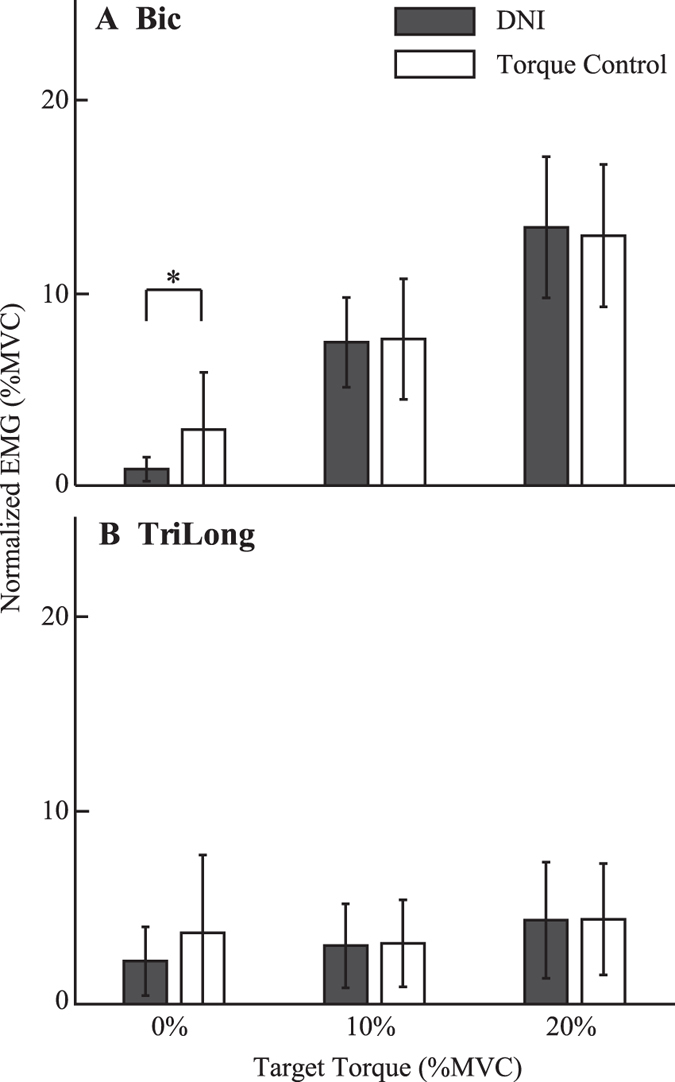
Comparison of the average EMG activities from Bic (**A**) and TriLong (**B**) in the DNI and the torque control tasks. The average EMG activities were quantified as the average amplitude of the rectified EMG in each trial, and then normalized to the MVC. Error bars indicate standard deviations. The asterisk *indicates significant difference at *P* < 0.05.

There was no significant difference between the average rectified EMGs measured in the torque control and DNI tasks performed when generating an active flexion torque about the elbow. This finding held for the elbow muscles at bias torques of 10% MVC (Biceps (Bic) *F*_1,9_ = 0.0, *P* = 0.99; Triceps Long Head (TriLong), *F*_1,9_ = 0.47, *P* = 0.51) and 20% MVC (Bic, *F*_1,9_ = 1.6, *P* = 0.23; TriLong, *F*_1,9_ = 0.04, *P* = 0.84) (Fig. 3). For 0% MVC, there was little muscle activation for both muscles in the DNI, suggesting that the subjects were at rest. In contrast, there was EMG activity in both muscles during the torque control task: EMG activity of Bic was significantly greater in the torque control task than that in the DNI task (*F*_1,9_ = 8.5, *P* = 0.02); there was also increase in the average TriLong activity, though this did not reach significance (*F*_1,9_ = 4.3, *P* = 0.07). This increase in Bic EMG suggests that subjects voluntarily activated their muscles in an effort to complete the 0% MVC torque control task, even though those efforts were not effective (e.g. Figs 1 B and 2A).

**Figure 4:**
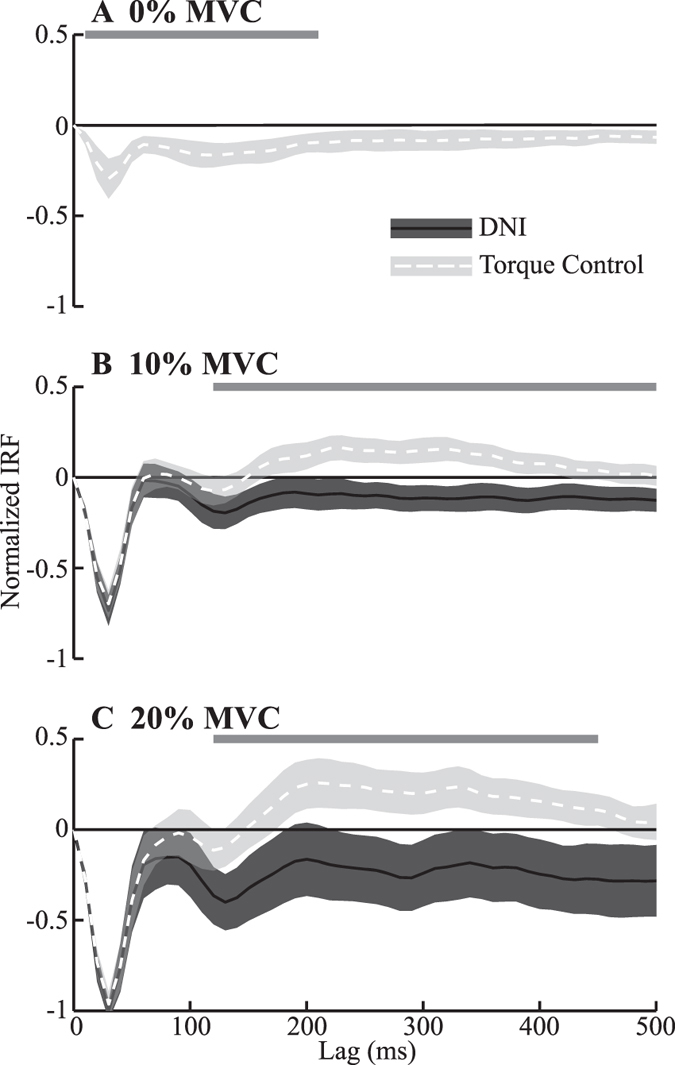
Group average of the normalized IRFs estimated between elbow joint acceleration and EMGs of Biceps (Bic) in the DNI (solid curves) and the torque control (dashed curves) tasks. (**A**,**B** and **C**) show IRFs for Bic at 0%, 10% and 20% MVCs, respectively. All the IRFs were normalized to the peak of the short-latency reflexes at 20% MVC. The bars on the top of each panel indicate the range of time lag in which the IRFs were significantly different between the torque control and the DNI tasks. The black horizontal lines in (**B** and **C**) indicates zero. The shaded areas indicate 95% confidence intervals.

There were task-dependent changes in the perturbation-evoked Bic EMG when generating volitional torques of 10% and 20% MVC. This was observed using impulse response functions (IRFs) to describe the causal relationship between joint acceleration and Bic EMG (Fig. 4B and C). These IRFs displayed a sharp peak at approximately 30 ms, a time associated with the latency of the short-latency stretch reflex in this muscle^29^. The amplitudes of these peaks did not differ between the DNI and torque control tasks performed at 10% MVC (*F*_1,9_ = 0.9, *P* = 0.36) or 20% MVC (*F*_1,9_ = 1.4, *P* = 0.26). There were noticeable differences between the IRFs estimated from the torque control and the DNI tasks after approximately 70 ms; these differences became statistically significant around 120 ms, and remained significant for almost the duration of the estimated IRFs (500 ms). The IRFs from the DNI tasks performed at 10% and 20% MVC had only negative values, suggesting a feedback response in opposition to the imposed joint movement. In contrast, the IRFs from the torque control tasks became positive after approximately 150 ms, suggesting a feedback response that assisted the imposed movement, thereby decreasing impedance. For the tasks performed at 0% MVC (Fig. 4A), IRFs of substantial magnitude only could be estimated for the torque control case, since the Bic EMGs were close to zero for the DNI trials. The IRFs for the 0% MVC torque control tasks remained negative, suggesting that the feedback under these conditions always served to oppose the perturbation. There were only small perturbation-evoked EMG activities in TriLong during 10% and 20% MVC trials (not shown), as our protocol for these trials only required activation of the Bic but not the TriLong. Similar to Bic, the estimated IRFs of TriLong at 0% MVC opposed the applied perturbation (not shown).

**Figure 5:**
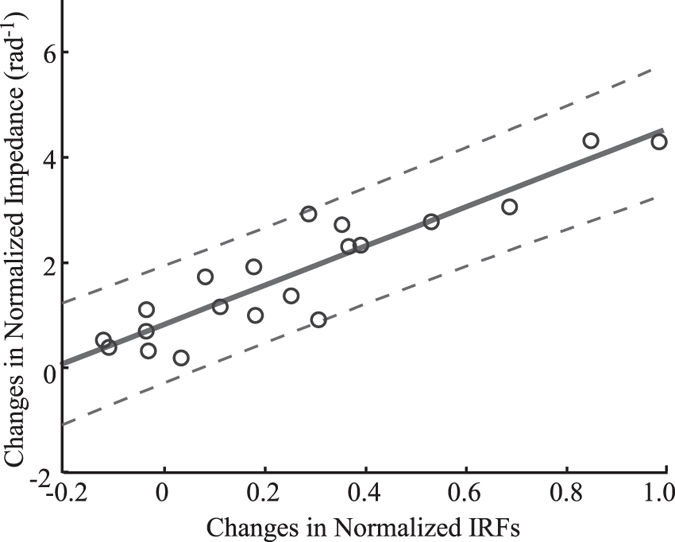
Linear regression between the average task-dependent changes in the normalized IRFs and the average task-dependent changes in the normalized impedance for Bic. The change in the normalized IRFs was averaged over lags ranging from 100–200 ms, and the change in the normalized low frequency impedance was averaged over the range from 0-1 Hz. Each circle corresponds to one subject at either 10% or 20% MVC. Thin dashed lines above and below the thick regression line define 95% confidence intervals (CIs).

The observed perturbation-evoked changes in muscle activity during the torque control tasks were correlated with the ability to regulate elbow impedance. There was a strong linear relationship between the task-dependent change in the normalized low frequency impedance and the corresponding change in the normalized IRFs in Bic for each subject (Fig. 5; r^2^ = 0.84, *P* < 0.0001). This relationship was not sensitive to the time period used to summarize the IRFs, yielding similar results for 100 ms ranges starting from 100 to 150 ms, or to the amount of data used to estimate the impedance transfer functions and IRFs; the latter was assessed by recomputing Fig. 5 with only 80% of the available data. Comparisons were not made at 0% MVC since there was negligible EMG during the DNI task at this torque level.

It is important to note that the averaged Variance Accounted For (VAF) of IRFs characterizing the relationship between joint motions and EMG ranged from only 12% to 17% for Bic during the 10% and 20% MVC trials. These values are lower than those reported by Kearney and Hunter^30^ for the muscles of the ankle, yet our results demonstrate that they are clearly related to the simultaneously observed changes in elbow impedance. We expect that the low VAFs in our experiment result from the fact that our IRFs were characterizing long periods of average sustained muscle activity, even though the instantaneous activity is a noisy process.

## Discussion

This study examined how well humans can maintain a voluntary joint torque when facing unpredictable perturbations of posture. This was achieved by estimating elbow impedance during DNI and torque control tasks. Our results demonstrated that individuals can reduce the low-frequency components of elbow impedance, thereby reducing the torque generated by unexpected perturbations. Analysis of the major elbow muscle EMGs (Bic and TriLong) elicited by the applied perturbations indicated that the observed reduction in elbow impedance during torque control could be attributed to a feedback regulation of muscle activity. The feedback response estimated in the two tasks began to differ at approximately 70 ms after the perturbation, and this difference became significant at approximately 120 ms. There was no difference in the short-latency reflex elicited during each task. Together, these results quantify the efficacy with which elbow torque can be regulated during postural tasks, and suggest that this behavior is mediated through changes in the volitional or possibly long-latency reflex responses to unexpected perturbations, but not through changes in the response from short-latency reflex pathways.

### Performance during torque control tasks

The active regulation of joint torque was characterized by a volitional decrease in joint impedance for frequencies below approximately 1 Hz (Fig. 2B and C). This frequency range is similar to that reported in human operator studies, in which it has been demonstrated that individuals can accurately track a randomly moving target below ~0.7 Hz, and that tracking becomes intractable for targets moving with a bandwidth above ~2 Hz^31–33^. These findings were also in general consistent with those of Mugge *et al*.^20, 24^, who showed lower ankle impedance in the torque control task than in the DNI. An interesting difference between our results and those of Mugge *et al*. is our finding that subjects were unable to actively reduce joint impedance in the 0% MVC trials below the passive impedance of the joint measured during the DNI task (Fig. 2A). In contrast, Mugge demonstrated that such a reduction was possible at the ankle. One possible explanation for this difference is that at rest, the passive impedance of the ankle at low frequencies (~30 Nm/rad^34^) is about ten times larger than the passive impedance of the elbow in the same frequency range (~3 Nm/rad, Fig. 2A). Therefore, it is possible that the intrinsic properties of the muscles^35^ limit the lowest impedance that can be achieved with active regulation to a value above that of the passive elbow impedance but below that of the ankle.

### Strategies for torque regulation

Subjects adopted a strategy of lowering elbow impedance to better regulate joint torques in our experiments. This strategy was proposed by Kolesnikov, *et al*.^18^ as one method for regulating force, though their experiments did not provide evidence for the use of this strategy. Instead, they observed an increase in the impedance of the arm when subjects were instructed to regulate force. The major difference between their experiment and ours is that subjects in Kolesnikov’s 2011 study were instructed to regulate force while the limb was perturbed with slow, sinusoidal perturbations that had a frequency of 0.125 Hz. In contrast to this predictable perturbation, our experiments employed faster stochastic perturbations with substantial power up to 1 Hz. Since the perturbations used by Kolesnikov *et al*. were easy to predict, their subjects may have gradually formed an internal model of the perturbation, and used position control to regulate force. The increased impedance observed in their experiments could therefore be explained by the need to producing accurate motions^14^. Since our experiments, as well as those used by Mugge, *et al*.^20, 24^ used unpredictable perturbations, only a reactive strategy could be used, resulting in the observed decreases in joint impedance.

### Mechanisms for lowering elbow impedance

Impedance was reduced through longer-latency changes in muscle activation that served to assist rather than resist the imposed perturbations. This conclusion is based on the finding that the IRFs relating elbow acceleration to EMG significantly differed in two tasks starting at a time lag of 120 ms (Fig. 4). In this time lag range, IRFs from the DNI task stayed negative, whereas those from the torque control crossed zero becoming positive. The positive components of the causal IRFs estimated from the torque control tasks indicate feedback responses that assist the imposed perturbations, thereby countering intrinsic muscle stiffness and reducing the net impedance of the muscles and joint. These differences in the IRFs estimated from the two tasks were strongly correlated with the corresponding differences in the impedance transfer function below 1 Hz (Fig. 5). This correlation also suggests that these long-latency feedback responses contributed to the ability to complete the torque control task.

Long-latency reflexes may have contributed to the observed results. These responses, typically occurring ~70 ms, have been shown to be lower in “maintain force” tasks than in “maintain position” tasks, and have a corresponding effect on impedance^21–23, 36^. In our results, the earliest observed task-dependent, perturbation-evoked changes in EMG occurred at ~70 ms. These changes, however, were not statistically significant until ~120 ms (Fig. 4). The lack of significance at 70 ms may result from the relatively low velocity perturbations—due to limited bandwidth of the full power part and the small amplitude of the reduced power—used in the present study, compared to the high velocity of the rapid transient perturbations typically used when studying reflex mechanisms^23, 29^.

Higher-level feedback responses may also have been important. The timing at which the IRF in two tasks started to differ significantly (~120 ms) is similar to the time needed to receive, process the proprioceptive information and then send neural commands to muscles^37^, suggesting the important contribution of proprioceptive feedback during the torque regulation task^24^. This timing was shorter than what is needed for utilizing the visual feedback, typically reported as at least 190 ms^38, 39^. But, since the difference of the IRFs between two tasks remained significant to ~500 ms, we certainly cannot rule out contributions from visual, as well as proprioceptive pathways to the observed impedance regulation. Future experiments that perturb vision as well as proprioception could be used to distinguish the compensations mediated by each of these systems.

Changes in short-latency reflexes were not a factor in our experiments. Mugge, *et al*.^20^ proposed that short-latency reflexes (~30 ms delay), especially those stemming from excitation of Golgi tendon organs, contributed to the task-dependent modulation of ankle impedance observed in their study. Those conclusions, however, were based on indirect assessments. Our results do not support such a mechanism, as the short-latency reflexes elicited in our protocols did not differ across tasks (Fig. 4). Our finding is consistent with previous studies examining reflex responses during force and position control in the upper limb^21–23^. It should be noted, however that short-latency reflexes can be altered over a longer periods of training^40–42^, and we cannot rule out the possibility that those changes could alter the ability to regulate force.

Our results also could not be explained by changes in average joint torque or muscle co-contraction. Though it has been well documented that joint impedance varies with joint torque^9–11, 13^, there were no significant differences in the average torques between two tasks performed at 10% and 20% MVC (Fig. 1C). Joint impedance also varies with co-activation of agonist and antagonist muscles^17, 43, 44^. However, average EMGs of major elbow muscles monitored in the experiment did not show significant differences between the torque control and DNI tasks performed at 10% or 20% of MVCs (Fig. 3). These findings support the role of feedback in the observed impedance regulation.

Our results are consistent with the optimal regulation of limb impedance, given the constraints related to delayed neural feedback and the increase of intrinsic muscle stiffness with increasing force. In optimal feedback control, as the goals of the tasks change, the central nervous system (CNS) modulates feedback responses to achieve task goals^45, 46^. In the current study, when the task was switched from DNI to torque control, subjects regulated torque by modulating feedback responses to assist rather than resist the imposed disturbances. The task-dependent feedback compensation became significant after ~120 ms, but started as early as ~70 ms after the externally imposed displacements. These results are consistent with those by Pruszynski, *et al*.^47^, showing that both voluntary responses and long-latency reflexes can be modulated according to task goals, though the latter is modulated to a lesser extent. These changes in feedback control of muscle activity may be achieved by the enhanced force feedback during torque control task compared with the DNI task^20^. We previously demonstrated that the experimental findings reported here are remarkably consistent with an optimal controller that regulates force feedback while considering physiological neural delays and realistic intrinsic muscle properties^48^.

For tasks involving force/torque control in multiple degrees of freedom, alternative strategies may be possible including those that selectively modulate impedance in task-relevant directions. A number of multi-joint studies have demonstrated that arm impedance can be increased in selective directions through preferential tuning of muscle activity^36, 49–51^, or changes in arm posture^52^. We expect that similar means could be used to lower impedance in task-relevant directions, as required for force/torque control. In the case of postural adaptations, this would often result high-impedance along task-irrelevant directions, analogous to the partitioning of motor variability observed in kinematic tasks^53^.

### Conclusions

Different tasks require different magnitudes of limb impedance. While most studies have focused on the ability to increase impedance as needed to maintain an accurate posture, few have assessed the ability to reduce impedance during tasks that require the accurate production of force. Our results demonstrate the limits on the ability to use feedback control for reducing impedance when generating significant volitional forces. In our task, this ability arose through physiological mechanisms having a delay of 70–120 ms, suggesting that the fastest spinal reflexes were not involved for the tasks and training periods used in our work. These findings suggest that changes to the systems responsible for feedback control, such as those that occur naturally with aging or as a result of trauma from stroke or other injuries^54–58^, may have a profound effect on the ability to regulate force in the upper limb, similar to what has previously been shown for the control of upright balance and posture^54, 59, 60^. Furthermore, quantifying these changes may ultimately provide a means to assess underlying changes that are not as readily apparent when examining the ability to regulate position.

## Methods

### Subjects

Ten subjects (7 men and 3 women) with an age range of 23–45, and no prior history of neurological disease or injury to the elbow, participated in this study. All experimental procedures were approved by the Institutional Review Board of Northwestern University (IRB protocol STU00009204), required informed consent and performed in accordance with the relevant guidelines and regulations.

### Equipment

**Figure 6:**
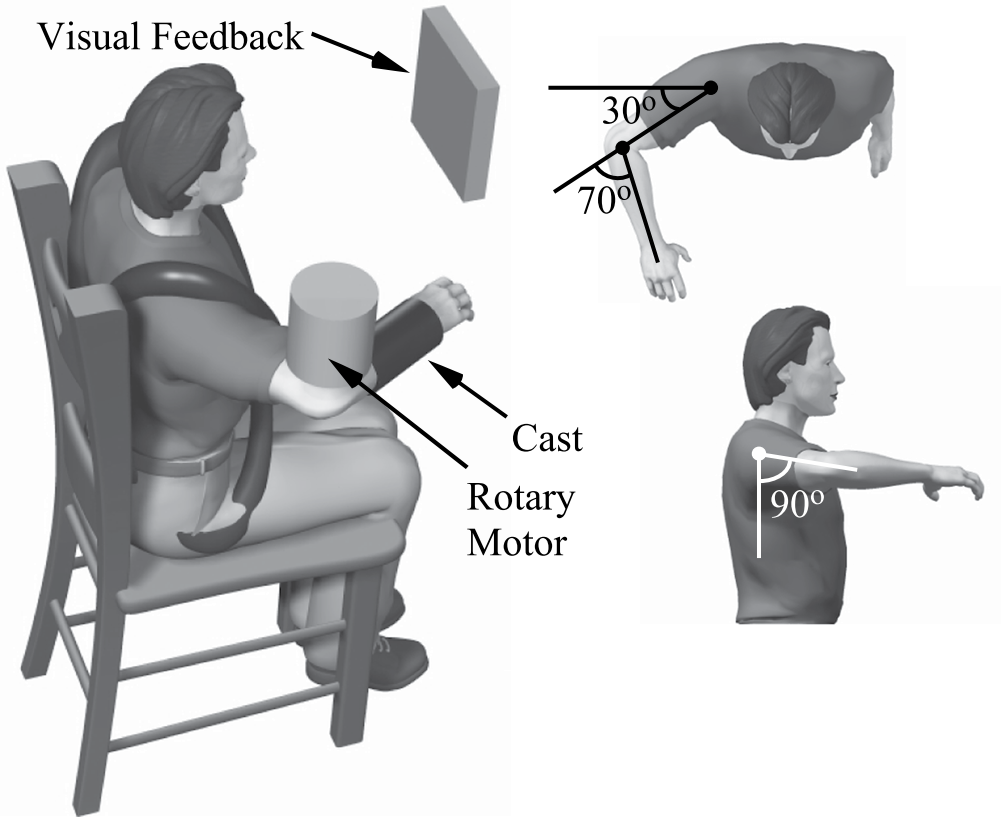
Experimental setup. The right forearm of each subject was positioned in the horizontal plane at a nominal posture of 90° shoulder abduction, 30° shoulder flexion and 70° elbow flexion with the forearm fully pronated. The wrist joint was immobilized in neutral position using a rigid custom-made plastic cast. The cast was attached to a rotary motor, aligned such that the motor axis was in line with the elbow flexion/extension axis. The computer-controlled rotary motor (BSM90N- 3150AX; Baldor Electric Company, Fort Smith, AR) was used to apply small angular position perturbations to the elbow joint. Visual feedback of joint torque was displayed to assist with task completion.

Subjects were comfortably seated in an adjustable chair (Biodex, NY), and movement of the trunk was minimized using a set of straps placed across the torso. The right forearm of each subject was positioned in the horizontal plane using a rigid custom-made plastic cast, which was attached to a rotary motor (Fig. 6). The rotary motor was used to apply small angular stochastic position perturbations to the elbow with an effective resolution of 6.3 × 10^−5^ rad. Through the whole experiment, the rotary motor was controlled as a rigid position servo with a stiffness of 35 kNm/rad. Forces and moments were measured using a six degree-of-freedom load cell (630N80; JR3, Inc, Woodland, CA), and the motor was controlled in real-time using the Matlab xPC.

Bipolar, surface electrodes (model #272; Noraxon USA, Scottsdale, AZ) were used to record surface electromyographic (EMG) activity in Biceps (Bic) and Triceps long head (TriLong). Standard skin preparation was used before applying each electrode. EMGs were amplified using a Bortec AMT-16 system (Bortec Biomedical, Calgary, AB, Canada) with a bandwidth of 10–1,000 Hz, an input impedance of 10 GΩ, and a common-mode rejection ratio of 115 dB at 60 Hz. The resulting analog signals were anti-alias filtered using custom-built, differential input, 5th-order Bessel filters with a cutoff frequency of 500 Hz and then sampled at 2,500 Hz with a 16-bit data acquisition system (PCI-DAS1602/16; Measurement Computing Corporation, Norton, MA).

### Protocol

Isometric MVCs were collected at the start of each experiment. These data were later used to normalize the EMGs recorded from each subject. Separate MVCs were recorded in flexion and extension, while the subject was attached to the rotary motor (Fig. 6).

**Figure 7:**
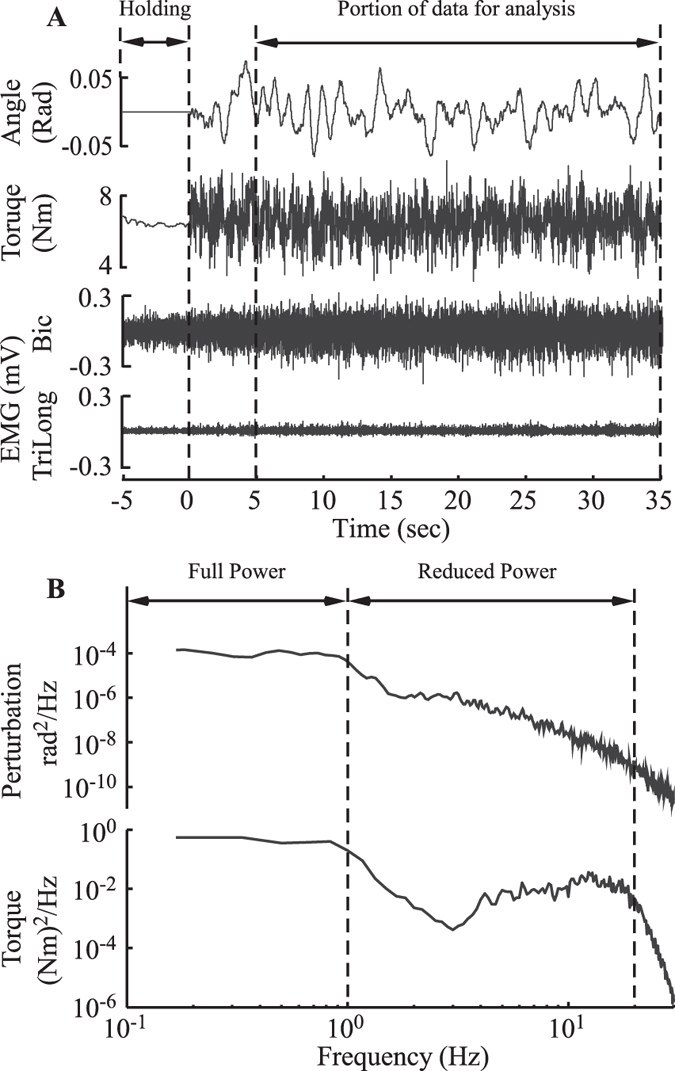
Typical experimental data. (**A**) shows the angular perturbation to the elbow, the subject’s elbow torque and elicited EMGs during perturbation. For each trial in both tasks, subjects were first instructed to exert a specified torque against rotary motor, and to maintain that torque for 5 seconds. After this time the perturbation commenced, lasting for 35 seconds. Only last 30 seconds data were analyzed. (**B**) shows the power spectra of the applied angular position perturbations and the resulted elbow torques. Due to the second order characteristics of the elbow^11, 62^, this 2-stage filtering process resulted in a reduced power torque spectrum that was approximately flat from 4–20 Hz.

Specific instructions were given about how to perform the DNI and the torque control tasks. Subjects were first instructed to exert and maintain a specified torque against rotary motor for 5 seconds, after which 35-second perturbation commenced (Fig. 7A). During the DNI task, subjects were instructed to keep the muscle activation the same as before the perturbation started, and not to react to the perturbation. For the torque control task, subjects were instructed to keep their torque constant during perturbation by voluntarily activating their muscles, as needed.

Subjects were provided with real-time visual feedback of elbow torque to assist the completion of two tasks. The visual feedback was scaled so that the variance of the displayed torque remained constant for all levels of voluntary torque generated by the subject. However, the visual feedback was filtered accordingly in each task to assist subjects in drastically different ways. In the torque control task, the visual feedback was filtered by a 2^nd^ order lowpass Butterworth filter with a cutoff frequency of 2 Hz. This filter was used to reduce the large torque transients induced by the perturbations while still providing subjects with feedback on a time-scale relevant to human operator tracking performance^31, 33^ so that subjects could compensate accordingly. This short-term error information was obscured in the DNI task by a 2^nd^ order lowpass Butterworth filter with a cutoff frequency of 0.1 Hz. The 0.1 Hz filtering was provided to prevent substantial drift from the desired target torque during the DNI task, but was not sufficient to allow subjects to compensate for errors resulting from individual perturbations, which had a “full-power” bandwidth of 1.0 Hz.

Each subject completed approximately one hour of training, during which subjects had become familiar with the tasks and had largely achieved consistent performance in both tasks. Testing was performed on a separate day. The testing session evaluated performance in the DNI and torque control tasks, each at three levels of target torque: 0%, 10% and 20% MVC, all in elbow flexion. Each of these 6 conditions was repeated three times, yielding a total of 18 trials. The trials were grouped into two randomized blocks according to task; within each task block, trials were randomized in terms of MVC level. Each trial was followed by a one-minute rest to avoid fatigue.

Stochastic displacement perturbations were used to estimate elbow impedance. These perturbations consisted of two components: a “full power” component that had a flat power spectra up to 1 Hz and an amplitude standard deviation of 1.5 degrees (0.026 rad), and a “reduced power” component that had an amplitude standard deviation of only 0.3 degree (0.0052 rad) but power up to 20 Hz (Fig. 7B). The two-component spectral characteristics of the perturbations were designed so that torque control behavior in the relatively low frequency domain can be elicited by the “full power” component, while the impedance can still be estimated in full bandwidth via the “reduced power” component, similar to the method used by^61^. The spectral characteristics of the “full power” component were obtained using a 5^th^ order lowpass Butterworth filter with a cutoff frequency of 1 Hz. This filter was applied to make sure that the bandwidth of the “full power” component was within the frequency range (0.7~1.2 Hz) that well elicits the torque control behavior^20, 33^. The reduced power component was obtained using a 2-stage filtering process. First, a 2nd order lowpass Butterworth filter with a cutoff frequency of 4 Hz was chosen so that the torque spectrum resulting from the imposed perturbations was approximately flat from 4-20 Hz, arising from the second order characteristics of the elbow impedance^11, 62^. Then, a second 4^th^ order lowpass Butterworth filter with a cutoff frequency of 20 Hz was applied since the resulted torque response above this frequency was mostly dominated by inertia, which was identical in the DNI and torque control tasks.

### Data analysis

**Figure 8:**
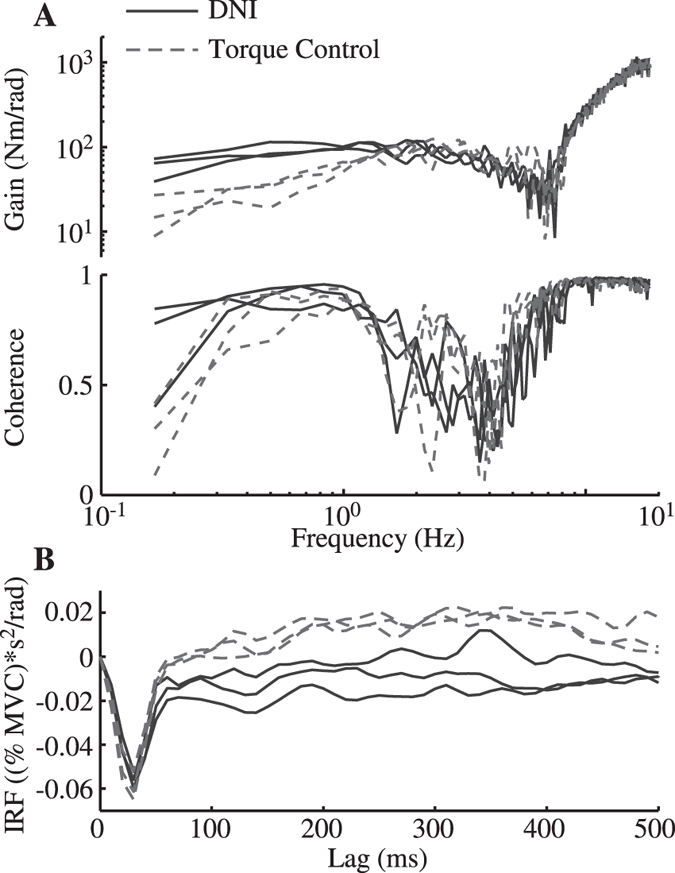
Typical estimated transfer functions and impulse response functions. (**A**) The estimated impedance transfer functions from subject S1 during the two tasks, when performed with a target torque of 20% MVC (16.7N). The gain (top panel) and coherence (bottom panel) are shown for the DNI (solid curves) and the torque control (dashed curves) tasks. Three repetitions are shown for each condition. (**B**) Estimated impulse response function (IRF) from joint acceleration to Bic EMG at 10% MVC from the same subject. Three repetitions of the IRF estimated from the DNI (solid curves) and the torque control task (dashed curves) are shown. Due to laboratory sign convention, negative regions of the IRF correspond to perturbation-evoked changes in EMG that resist the accelerating joint; positive regions denote perturbation-evoked changes in EMG that provide assistance.

The ability to maintain a constant torque in the presence of stochastic perturbations was assessed by comparing the standard deviation of the measured torque, and the elbow impedance estimated during the DNI and torque control tasks. All analyses were performed on the final 30 seconds of collected data to avoid possible adjustments in subject’s effort at the start of each trial. Nonparametric system identification^63^ was used to estimate the elbow impedance transfer functions, as well as the coherence between the measured displacements and torques. The transfer function was used to quantify the magnitude of the elbow impedance as a function of frequency; coherence was used as a measure of how well this model characterized the measured responses^64^. Both were computed using 6-second data windows and an overlap of 50%, resulting in a frequency resolution of 0.167 Hz (Fig. 8A). The estimated transfer functions for the active tasks were normalized by the subject-specific target torques at 10% and 20% MVCs to facilitate comparisons across subjects, but not at 0% MVC because of zero target torque. Note that the rotary motor was configured more than 2 orders of magnitude stiffer than the human elbow during these experiments, allowing open-loop system identification methods to be used^65^. The estimated nonparametric elbow impedance transfer functions were not further parameterized by a second order system including stiffness, viscosity, and inertia, because subjects’ intervention in the torque control task induced nonstationary elbow mechanics that cannot be appropriately described by a second order system (Fig. 2).

EMGs were processed by first removing the mean value, rectifying, and then normalizing to the EMGs recorded during MVCs. The normalization constant corresponded to the maximum average rectified EMG sustained for 0.5 second during the MVC trials. After this initial processing, the EMGs and the recorded positions and torques were digitally anti-alias filtered and resampled at 100 Hz before further processing. No substantial effects of fatigue were observed in the collected EMG data, as evidenced by stationary amplitudes over the course of each trial.

EMGs were used to evaluate changes in the neural control of muscle activation during the two tasks. Changes in average muscle activation were quantified by the average rectified EMG within each trial, providing an estimate of the steady-state feedforward control to the muscles. Changes in the feedback regulation of muscle activation were assessed by estimating the nonparametric impulse response function (IRF) between the elbow joint acceleration and the rectified EMGs^63^ (Fig. 8B). The acceleration was used as the kinematic variable relating the arm movement and EMG activity since it allowed for the simplest interpretation of the IRF. This allowed us to identify the changes in muscle activity that resulted from changes in joint acceleration, thereby indicative of a feedback response. The estimated IRFs contained a sharp peak at approximately 30 ms, likely corresponding to the short latency reflex response to the perturbations^29^. The magnitude of this peak at 20% MVC was used to normalize estimated IRFs at all three tested MVC levels, thereby facilitating comparison across subjects.

The sign of the estimated IRF indicates whether the responses of EMGs were resisting or assisting the applied perturbation. The interpretation of the sign of the IRF for our data depends on two specific factors: (i) all target torques were in flexion, and (ii) our laboratory convention dictates that positive (negative) changes in elbow angle correspond to elbow flexion (extension), which shortens (lengthens) the elbow flexor muscles, such as the Bic. Thus, a negative component of the IRF from elbow acceleration to Bic EMG would suggest a resisting response, which could result from either an increase in muscle activation in response to muscle lengthening or a decrease in activation in response to shortening. Such changes would increase the impedance of the joint. In contrast, a positive component of the same IRF would suggest an assistive response, leading to a decrease in elbow impedance, as would be appropriate for the torque control task.

### Statistical analysis of experimental data

We hypothesized that the low frequency component of elbow impedance would be smaller in the torque control task than in the DNI task. This hypothesis was tested at each frequency point up to 10 Hz using linear mixed effect models with subjects as a random factor. Analysis of Variance (ANOVA) was used to identify significant effects. Separate analyses were performed for each level of background torque. Decreased impedance should also result in a corresponding decrease in the torque variation measured during the torque control task. Thus, the same statistical analysis was used to test whether there were task-dependent changes in the standard deviation of the torque measured in each task.

Additional statistical analyses were performed to elucidate possible mechanisms contributing to the observed differences in elbow impedance. Specifically, we were interested in quantifying changes in muscle activation within each task. The average rectified EMG within each trial was used to assess if there were changes in the net muscle activity across tasks. Differences across tasks were evaluated using a linear mixed effect model with task (DNI or torque control) as a fixed factor and subject as a random factor. Differences in the estimated IRFs describing the relationship between elbow acceleration and the rectified EMG within each muscle were evaluated using the same statistical analysis, conducted at each of the estimated lags from 0–500 ms (10 ms resolution).

Linear regression was used to assess the potential relationship between the task-dependent change in the low frequency impedance and the corresponding change in the IRFs describing the perturbation-evoked EMG in Bic for each subject. The change in the normalized low frequency impedance was averaged over the range from 0-1 Hz, since significant task-dependent differences in impedance were detected within this range (see Results, Fig. 2B and C). The change in the normalized IRFs was averaged over lags ranging from 100–200 ms, the earliest range within which the IRFs began to show significant differences between two tasks (see Results, Fig. 4B and C). Data from the 10% and 20% MVC trials were combined for all subjects. All statistical analyses were performed in MATLAB. Statistical significance was tested at the level of 0.05.
